# An Analytical Model for Estimating the Bending Curvatures of Metal Sheets in Laser Peen Forming

**DOI:** 10.3390/ma14020462

**Published:** 2021-01-19

**Authors:** Yunxia Ye, Zeng Nie, Xu Huang, Xudong Ren, Lin Li

**Affiliations:** 1 School of Mechanical Engineering, Jiangsu University, Xuefu Road, Zhenjiang 212013, China; 2221803108@stmail.ujs.edu.cn (Z.N.); 2221903105@stmail.ujs.edu.cn (X.H.); renxd@ujs.edu.cn (X.R.); 2 Institute of Micro-Nano Optoelectronics and Terahertz Technology, Jiangsu University, Xuefu Road, Zhenjiang 212013, China; 3 Laser Processing Research Centre, School of Mechanical, Aerospace and Civil Engineering, The University of Manchester, Manchester M13 9PL, UK; lin.li@manchester.ac.uk

**Keywords:** laser peen forming, curvature, analytical model

## Abstract

Laser peen forming (LPF) is suitable for shaping sheet metals without the requirement for die/mold and without causing high temperatures. An analytical model for estimating the bending curvatures of LPF is convenient and necessary for better understanding of the physical processes involved. In this paper, we describe a new analytical model based on internal force balance and the energy transformation in LPF. Experiments on 2024 aluminum alloy sheets of 1–3 mm thickness were performed to validate the analytical model. The results showed that for 1 mm and 3 mm thick–thin plates, the curvature obtained by the analytical model changes from −14 × 10^−4^ mm^−1^ and −1 × 10^−4^ mm^−1^ to 55 × 10^−4^ mm^−1^ and −21 × 10^−4^ mm^−1^, respectively, with the increase of laser energy, which is consistent with the experimental trend. So, when either the stress gradient mechanism (SGM) or the shock bending mechanism (SBM) overwhelmingly dominated the forming process, the analytical model could give relatively accurate predicted curvatures compared with the experimental data. Under those conditions where SGM and SBM were comparable, the accuracy of the model was low, because of the complex stress distributions within the material, and the complex energy coupling process under these conditions.

## 1. Introduction

Laser peen forming (LPF) has attracted a great amount of attention from both industry and academia in recent years due to its technological advantages. Comparing with traditional die-/mold-based forming and laser hot forming, it has the advantages of being die-/mold-free and noncontact, and has the further advantages of capability of forming thick and large sheets and production of complex geometry without generating heat-affected zones. Typical applications of LPF include forming of aircraft skins, wings, nose wings of bullet trains, and rocket fuel tanks.

The first report of laser peen forming was in 2002 by Hackle et al. [1], who proposed that the technology was especially suitable for forming sheets with a thickness greater than 3/4 inches that were difficult to form with traditional methods. Zhou et al. [2] investigated key process parameters on the convex bending of 6061-T6 aluminum alloy. They observed that the obtained plate arc height varied with number of laser shocks and the plate thickness. Wang et al. [3] conducted LPF experiments on 100 μm copper foils and found that the foil curvatures could evolve from concave to convex by changing the laser intensity from 3.57 GW/cm^2^ to 4.95 GW/cm^2^. The top and bottom surface residual stresses under these two conditions were also different. The factors that would affect the component geometry include: inertia after the laser peening forming, bending moment, and induced compressive stress. Hu et al. [4] observed both concave and convex curvature formation in laser peen forming of aluminum sheets of 0.5 mm to 2.25 mm in thickness. They observed that convex deformation was mainly due to stress gradient mechanism (SGM), while shock bending mechanism (SBM) was responsible for concave deformation. A three-dimensional numerical model was developed by them to simulate laser induced stress for understanding the mechanisms involved [5]. Zhou et al. [6] conducted numerical simulation based on finite element modeling (FEM) using ABAQUS and investigated the characteristics of residual stresses before and after laser peen forming. Yu et al. [7] developed an analytical model for the convex bending of laser shock forming with a uniform rectangular laser beam spot based on the biharmonic equations.

So far, many scholars have studied the law of laser shot peening and explored their application [8,9,10,11], but seldom do they conduct theoretical research on numerical analysis of the bending curvature of the sheet after laser shot peening. Although experiments and numerical simulations are helpful for understanding the behavior of the laser peen forming process and key factors affecting the geometry, an analytical model would provide more insight into the fundamental physical processes taking place in the process. At the same time, for engineering applications, there is an urgent need to construct a curvature analytical formula to more conveniently predict the bending results of the plate before the experiment or even the actual production application.

In this paper, we present a new analytical model for the prediction of both convex and concave deformation of metallic sheets through laser peen forming. Experiments were conducted to validate the model, and a detailed discussion of the process physical phenomena and mechanisms is given.

## 2. Mechanisms of Laser Peen Forming

**Figure 1:**
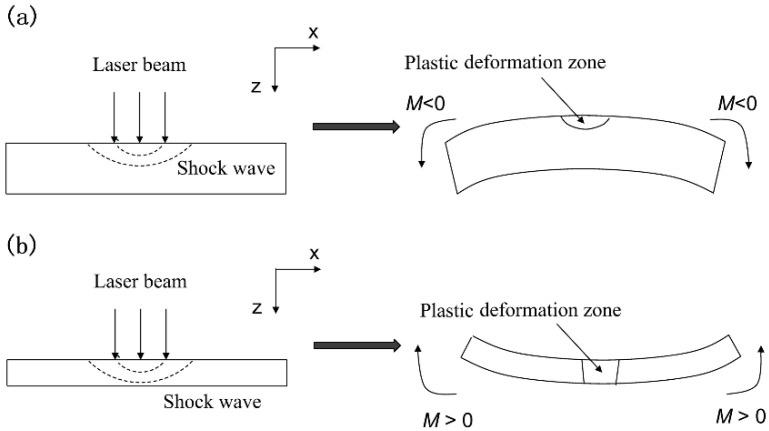
Bending mechanisms of laser peen forming: (**a**) SGM (**b**) SBM.

There exist two possible material deformations in laser peen forming [4], as illustrated in Figure 1. When the target sheet is thick or the laser intensity is moderate, the laser-induced shock wave can only induce plastic deformation and compressive residual stress in a thin layer of the target surface and elastic deformation is developed beneath the plastic deformation zone, which will cause a negative moment *M*, as shown in Figure 1a. Under this condition, the sheet will be bent in a convex mode. This bending mechanism is called the stress gradient mechanism (SGM). When the target sheet is thin or laser intensity is sufficiently high, a laser-induced shock wave can transmit through the overall sheet thickness, causing a positive moment *M*. The sheet will be bent in a concave mode, shown in Figure 1b. This mechanism is called the shock bending mechanism (SBM).

## 3. Theoretical Formulation

### 3.1. Convex Bending

**Figure 2:**
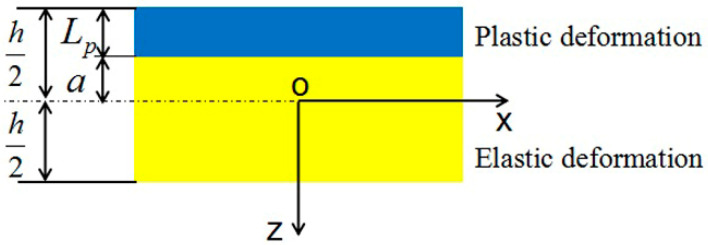
An illustration of deformation scheme for convex bending in laser peen formation.

In this case, laser-induced compressive stress and plastic deformation are confined within a thin layer beneath the top surface. The material below the surface plastic deformation zone is subjected to an elastic deformation. Thus, we divide the sheet into two layers along the thickness direction: a surface plastic deformation zone and a lower elastic deformation zone. As shown in Figure 2, under the laser-induced shock impact, the surface material is compressed to undergo a compressive strain along the *z* axis. If the volume compression of the solid target is not considered, the strain parallel to the surface will extend in the transverse direction. In the lower layer zone, due to the deformation compatibility, the material will undergo elastic strain and stress. In formulating the process relationships, the following assumptions are made: (1) disregarding the laser-induced compressive stress gradient within the upper layer. This is to assume that the compressive stress along the depth is uniform, and (2) the planes perpendicular to the middle layer of the sheet stay perpendicular to it during bending.

Since there is no additional external mechanical force involved after laser peen forming, the in-plane force and the corresponding bending moment resulted from the stress should be self-equilibrium. Therefore, we have,

(1) ∫σ(z)dz=0

(2) ∫zσ(z)dz=0

where *σ* is the stress developed, and *z* is the distance in the *z* direction. According to assumption (2), the normal strain *ε* in the *x* axis direction at any coordinate points (*x*, *z*) can be expressed as the sum of the plane strain, *ε*_0_, and the bending strain, *zk*_01_:

(3) $$\varepsilon =\varepsilon_{0}+zk_{01}$$

where *k*_01_ is the curvature of the middle layer.

Combining Equations (1) and (2) gives:

(4) $$-\int_{-\frac{h}{2}}^{-a}k\sigma_{ym}dz+E\int_{-a}^{\frac{h}{2}}\left(\varepsilon_{0}+zk_{01}\right)dz=0$$

(5) $$-\int_{-\frac{h}{2}}^{-a}zk\sigma_{ym}dz+E\int_{-a}^{\frac{h}{2}}z\left(\varepsilon_{0}+zk_{01}\right)dz=0$$

where *σ_ym_* is the static yield strength of sheet material, *k* is the average stress coefficient, *E* is the elastic modulus of the metal material, and *h* is the thickness of the metal sheet. The zone between [−*h*/2, −*a*] is subject to plastic deformation, and the zone between [−*a*, *h*/2] is elastic, as shown in Figure 2. The depth of the plastic zone, *L_p_*, is

(6) $$L_{P}=\frac{h}{2}a$$

Combining Equations (4)–(6), we have the convex curvature:

(7) $$k_{01}=-\frac{6k\sigma_{ym}}{E}\frac{hL_{P}}{{\left(h-L_{P}\right)}^{3}}$$

From Equation (7), with the appropriate values of *k* and *L_p_*, *k*_01_ can be derived.

#### 3.1.1. Plastic Zone Length, *L_p_*

The length of plastic zone, *L_p_*, is related to the laser peen forming parameters through the following relationships [12,13].

(8) $$L_{P}=\frac{C_{e}C_{P}\tau_{1}}{C_{e}-C_{P}}\frac{P_{max}-HEL}{2HEL}$$

where

(9) $$C_{e}=\sqrt{\frac{1-v}{\left(1+v\right)\left(1-2v\right)}\frac{E}{\rho }}$$

(10) $$C_{P}=\sqrt{\frac{1}{3\left(1-2v\right)}\frac{E}{\rho }}$$

(11) $$HEL=\frac{1-v}{v-2v}\sigma^{dym}$$

*C_e_* is elastic wave velocity, *C_p_* is plastic wave velocity, *τ*_1_ is the pressure pulse duration, *P_max_* is the maximum pressure of laser-induced shock wave, and *HEL* is Hugoniot elastic limit. *ν* is Poisson’s ratio and *σ^dym^* is dynamic yield strength of the metal material, which is two to four times larger than static yield strength, *σ_ym_*, under strong shock loading [14]. For the confined configuration during laser shock processing, *τ*_1_ is 2–3 times longer than laser pulse duration [15]. *P_max_* can be estimated by [16].

(12) $$P_{max}=0.01\sqrt{\frac{\alpha }{2\alpha +3}}\sqrt{Z}\sqrt{I}$$

and

(13) $$\frac{2}{Z}=\frac{1}{Z_{1}}+\frac{1}{Z_{2}}$$

(14) $$I=\frac{E_{Laser}}{\pi R^{2}\tau }$$

where *Z*_1_ and *Z*_2_ are the shock impedance of the confined medium and target material, respectively, and the unit is g/cm^2^·s. *α* is the interaction efficiency, and *E_Laser_* is the energy of laser pulse. *αE_Laser_* contributes to the pressure increase. *R* is the laser spot radius and *τ* is the laser pulse duration, and the unit of *I* is GW/cm^2^.

#### 3.1.2. Average Stress Coefficient *k*

If disregarding the disturbed condition, such as the influence of the reflected wave from the sample boundaries, the target surface will be subjected to the highest compressive residual stress because laser-induced shock wave attenuates rapidly in the thickness direction. In our analytical model, we simplify the stress distributions within the upper plastic zone to assume that they are uniform. We approximately take *σ_top_*/2 as the average stress along the depth within the plastic zone. *σ_top_* is the surface residual stress. Thus, the coefficient *k* is given by

(15) $$K=\frac{\sigma_{top}}{2\sigma_{ym}}$$

To validate the model, 2024 aluminum alloy was chosen as the target material, and water was used to act as the confining layer. Therefore, *Z*_1_ = 0.148 × 10^6^ g/cm^2^·s, *Z*_2_ = 1.506 × 10^6^ g/cm^2^·s, *σ_ym_* = 290 MPa, *E* = 72 GPa and *ν* = 0.34. In our experiments, the laser energy ranged from 2 J to 6 J. The laser pulse length, *τ* was about 20 ns, *R* = 1.5 mm, and *α* = 0.2. Under these conditions, the target sheets of the thicknesses 2 mm and 3 mm had convex bending curvatures. We measured the surface residual stresses of these samples. The maximum amplitude of compressive residual stress for the 2 mm thickness sheet at 2 J laser pulse energy was about 60 MPa. For laser energy 5 J, the surface compressive residual stress was about 70 MPa. According to Equation (15), the average stress coefficients were 0.1 and 0.12, respectively. For a sheet of 3 mm thickness, the maximum amplitude of compressive stress induced at 4 J laser energy was about 80 MPa, and at 6 J, it was about 100 MPa. According to Equation (15), the stress coefficients were about 0.137 and 0.172. In Zhou’s work [6], the maximum amplitude of the compressive stress for a 2 mm 2024 aluminum alloy sheet after bending at laser energy of 5.6 J was about 50 MPa, and it was about 80 MPa for a 3 mm plate. Hence, the average stress coefficients were 0.086 and 0.137, respectively. Combining the results of our experiments and Zhou’s results, the average stress coefficient ranged between 0.08~0.018 at laser energies ranging from 2 J to 6 J. So, in this work, we take the average stress coefficient *k* = 0.1. It should be noted that, although 2024 aluminum alloy is chosen as the target material in our work, if one chooses other materials as the target, the average stress coefficient *k* can also be taken in the same way.

### 3.2. Concave Bending

The bending mechanism of the concave deformation is significantly different from that of convex deformation. When the sheet thickness is small or laser pulse energy is large enough, laser-induced shock wave does not attenuate so seriously along the depth direction. In this case, SBM dominates the bending mechanism, and the analytical model based on SGM does not work. We therefore developed an analytical model for concave bending based on the energy transformation. Based on plate plastic deformation under a normal projectile impact [17], the following assumptions are made: (1) the kinetic energy of the sheet is entirely transformed into its strain energy, (2) there is no plane distortion between the planes during laser shock forming, i.e., the volume of sheet is constant during laser peen forming, (3) the material is linear work hardening, and (4) disregarding the coupling effects among laser impacts, that is to say, the first laser impact determines the sheet curvature, and the following laser impacts only bend other zones to this curvature.

**Figure 3:**
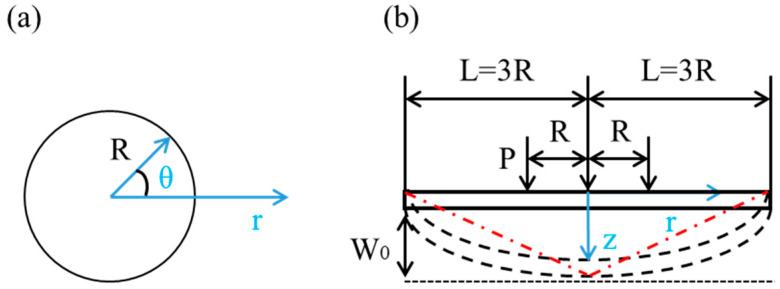
Schematic of concave deformation in laser shock forming. (**a**) polar coordinates (**b**) concave deformation.

In this case, we apply a cylindrical coordinate system. As shown in Figure 3, the laser shocked surface acts as a polar plane. The laser spot center is the origin and the thickness direction is the *z* axis.

The plastic strain energy of the plate is *E_p_*, and the elastic strain energy is *E_e_*. The shock wave induced kinetic energy of plate is *E_k_*. Then, as we assumed that the kinetic energy of plate is transformed into the strain energy of plate entirely,

(16) $$E_{p}+E_{e}=E_{k}$$

**(1)**  **Formulations of *E_p_* and *E_e_***

Their differential expressions are:

(17) $$dE_{P}=\overset{}{\underset{\Omega_{1}}{\int }}\left(\sigma_{r1}d\varepsilon_{r1}+\sigma_{\theta 1}d\varepsilon_{\theta 1}\right)d\Omega_{1}$$

(18) $$dE_{e}=\overset{}{\underset{\Omega_{2}}{\int }}\left(\sigma_{r2}d\varepsilon_{r2}+\sigma_{\theta 2}d\varepsilon_{\theta 2}\right)d\Omega_{2}$$

where *σ* and *ε* are the stress and strain, respectively. Subscripts *r* and *θ* refer to the radial and circumferential directions. Ω_1_ represents the volume of the plastic zone and Ω_2_ represents that of the elastic zone. The deflection of the plate under the impact of laser is *w*(*r*), so the radial strain and the circumferential strain can be expressed as:

(19) $$\varepsilon_{r1}=\frac{1}{2}{\left(\frac{dw}{dr}\right)}^{2}$$

(20) $$\varepsilon_{\theta 1}=\varepsilon_{\theta 2}=0$$

Yield condition can be expressed as:

(21) $${\left(\sigma_{r1}-\sigma_{\theta 1}\right)}^{2}+{\left(\sigma_{\theta 1}-\sigma_{z1}\right)}^{2}+{\left(\sigma_{z1}-\sigma_{r1}\right)}^{2}+6\left(\tau_{r\theta }^{2}+\tau_{\theta z}^{2}+\tau_{zr}^{2}\right)=2\sigma_{d}^{2}$$

According to assumption (2), we have the normal stress *σ_z_* = 0, normal strain *ε_z_* = 0 and shear strains (*τ_rθ_*, *τ_θz_* and *τ_zr_*) are all 0; then, the yield condition becomes

(22) $$\sigma_{r1}{}^{2}-\sigma_{r1}\sigma_{\theta 1}+\sigma_{\theta 1}^{2}=\sigma_{d}^{2}$$

According to assumption (3), the stress in elastic stage can be expressed as:

(23) σ=Eε

where *σ* is the elastic stress, *E* is the material elastic modulus, and *ε* is the elastic strain. The stress in plastic stage can be expressed as:

(24) $$\sigma_{d}=\sigma_{ym}+E^{P}\varepsilon_{r1}$$

where *σ_d_* is plastic stress, *σ_ym_* is the static yield strength of the metal material, *E^P^* is tangent modulus, and *ε_r_*_1_ is plastic strain.

Combining Equations (22) and (24), we have:

(25) $$\sigma_{r1}{}^{2}-\sigma_{r1}\sigma_{\theta 1}+\sigma_{\theta 1}^{2}={\left(\sigma_{ym}+E^{P}\varepsilon_{r1}\right)}^{2}$$

According to the relationship between the stress and strain during the elastic stage, we have *σ_θ_*_1_ = *νσ_r_*_1_, where *ν* is Poisson’s ratio of the metal material. Then, simplifying Equation (25) gives:

(26) $$\sigma_{r1}=\frac{\sigma_{ym}+E^{P}\varepsilon_{r1}}{\sqrt{1-v+v^{2}}}$$

Combining Equations (17) and (26) gives

(27) $$dE_{P}=\overset{}{\underset{\Omega }{\int }}\frac{\sigma_{ym}+E^{P}\varepsilon_{r1}}{\sqrt{1-v+v^{2}}}d\varepsilon_{r1}d\Omega_{1}$$

If the plastic deformation zone does not transmit through the overall target thickness, a small layer of elastic deformation for concave bending will still exist. Then, we define the stress of elastic deformation zone as *σ_r_*_2_ = *kσ_ym_*, where *k* is average stress coefficient within this layer along the depth direction. Therefore, the elastic strain energy can be expressed as

(28) $$dE_{e}=\overset{}{\underset{\Omega_{2}}{\int }}\left(\sigma_{r2}d\varepsilon_{r2}+\sigma_{\theta 2}d\varepsilon_{\theta 2}\right)d\Omega_{2}=\overset{}{\underset{\Omega_{2}}{\int }}k\sigma_{ym}d\varepsilon_{r2}d\Omega_{2}$$

The differentials of Ω_1_ and Ω_2_ can be expressed as

(29) $$d\Omega_{1}=2\pi L_{P}rdr$$

(30) $$d\Omega_{2}=2\pi \left(h-L_{P}\right)rdr$$

where *h* is the thickness of target, and *L_p_* is the depth of plastic zone. *dε_r_*_1_ integrates from 0 to *ε_r_*_1_, and *dε_r_*_2_ integrates from 0 to *σ_ym_*/*E*. Hence, we have

(31) $$E_{P}=\frac{2\pi L_{pP}}{\sqrt{1-v+v^{2}}}\int_{0}^{L}\left(\sigma_{ym}\varepsilon_{r1}+\frac{1}{2}E^{P}\varepsilon_{r1}{}^{2}\right)rdr$$

(32) $$E_{e}=\frac{k\sigma_{ym}^{2}\pi R^{2}\left(hL_{P}\right)}{E}$$

where *L* represents the transverse size of the deformation area of plate after laser peen forming, as shown in Figure 3.

**(2)**  **Formulation of *E_k_***

**Figure 4:**
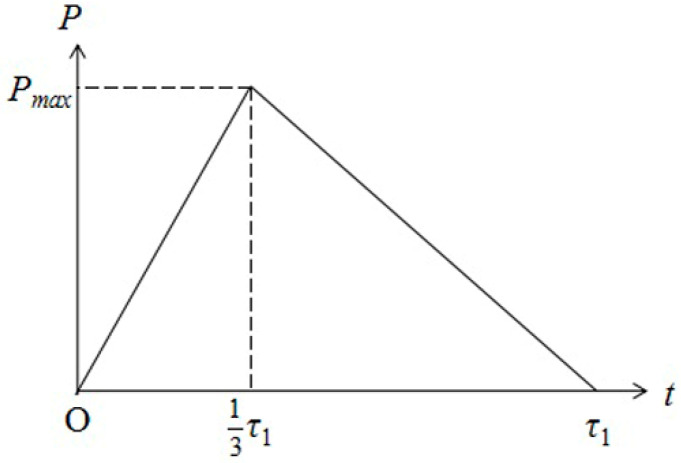
Schematic of laser-induced shock wave pressure vs. time.

Here, the shock wave is simplified as a triangular wave, as shown in Figure 4. Pulse duration of laser-induced shock wave is *τ*_1_. According to the measurement of laser-induced shock wave duration by Fabbro [16], we assume that the pressure reaches the maximum value *P_max_* at *τ*_1_/3. *P*_1_ represents the pressure within the rising stage and *P*_2_ the pressure within the declining stage.

According to Figure 4, *P*_1_ can be expressed as follows

(33) $$P_{1}=\frac{3P_{max}}{\tau_{1}}t$$

*P*_2_ can be expressed as

(34) $$P_{1}=\frac{3P_{max}}{2\tau_{1}}\left(t-\tau_{1}\right)$$

Then according to the definition of the impulse, the impulse caused by the shock wave on the plate can be obtained.

(35) $$\begin{array}{c} I=\int F\;dt=\int_{0}^{\tau_{1}}\int_{0}^{R}P\cdot 2\pi r\;drdt\;\\ =\int_{0}^{\frac{1}{3}\tau_{1}}\int_{0}^{R}P_{1}\cdot 2\pi r\;drdt+\int_{\frac{1}{3}\tau_{1}}^{\tau_{1}}\int_{0}^{R}P_{2}\cdot 2\pi r\;drdt\\ =\frac{1}{2}\tau_{1}\cdot \pi \cdot R^{2}\cdot P_{max} \end{array}$$

where *R* is the radius of the laser spot.

According to the experimental observation, we use line approximation to describe the deflection contour of plate after bending (shown with red dot-dashed line in Figure 3), expressed approximately as follows,

(36) $$w\left(r\right)=w_{0}\left(1-\frac{r}{L}\right)$$

in which *w*_0_ is the maximum deflection at the laser spot center, as shown in Figure 3. Then the expression of the point moving velocity *v*(*r*) along the *z* axis on the plate during laser peen forming can be obtained as accordingly:

(37) $$v\left(r\right)=v_{0}\left(1-\frac{r}{L}\right)$$

Combining the definition of the momentum and Equation (37), we have

(38) $$I=\int v\left(r\right)dm=\rho \int_{0}^{h}\int_{0}^{2\pi }\int_{0}^{L}v\left(r\right)rdr=2\pi \rho hv_{0}\int_{0}^{L}\left(1-\frac{r}{L}\right)rdr=\frac{1}{3}\pi \rho hv_{0}L^{2}$$

So, *v*_0_ can be expressed as

(39) $$v_{0}=\frac{3I}{\pi \rho hL^{2}}$$

Then, we have the kinetic energy expression of plate as:

(40) $$E_{k}=\int \frac{1}{2}v{\left(r\right)}^{2}dm=\rho \int_{0}^{h}\int_{0}^{2\pi }\int_{0}^{L}\frac{1}{2}v{\left(r\right)}^{2}rdr=\pi \rho h\int_{0}^{L}v_{0}{}^{2}{\left(1-\frac{r}{L}\right)}^{2}dr=\frac{3}{4}\frac{I^{2}}{\pi \rho hL^{2}}$$

**(3)**  **Formulation of the concave curvature *k*_02_**

From Equations (16), (31), and (32), we have the following relation

(41) $$\int_{0}^{L}{\left(\frac{dw}{dr}\right)}^{4}rdr+\frac{4\sigma_{ym}}{E^{P}}\int_{0}^{L}{\left(\frac{dw}{dr}\right)}^{2}rdr+\frac{4k\sigma_{ym}^{2}R^{2}\left(h-L_{P}\right)\sqrt{1-V+V^{2}}}{E\cdot E^{P}\cdot L_{P}}-\frac{4\sqrt{1-V+V^{2}}}{E^{P}\cdot L_{P}\cdot \pi }E_{k}=0$$

According to Equation (36), we have

(42) $$\int_{0}^{L}{\left(\frac{dw}{dr}\right)}^{4}rdr=\frac{w_{0}^{4}}{2L^{2}}$$

(43) $$\int_{0}^{L}{\left(\frac{dw}{dr}\right)}^{2}rdr=\frac{w_{0}^{2}}{2}$$

Considering Equations (40)–(43),we have

(44) $$w_{0}^{4}+\frac{4\sigma_{ym}L^{2}}{E^{P}}w_{0}^{2}+\frac{8k\sigma_{ym}^{2}R^{2}L^{2}\left(h-L_{P}\right)\sqrt{1-V+V^{2}}}{E\cdot E^{P}\cdot L_{P}}-\frac{6I^{2}\sqrt{1-V+V^{2}}}{\pi^{2}\cdot E^{P}\cdot L_{P}\cdot \rho \cdot h}=0$$

Through Equation (44), *w*_0_ can be solved. According to the geometric relation of an arc, one can obtain the curvature of the bending plate obtained through laser shock forming:

(45) $${\left(\dot{R}-w_{0}\right)}^{2}+L^{2}={\dot{R}}^{2}$$

(46) $$k_{02}=\frac{1}{\dot{R}}$$

(47) $$k_{02}=\frac{1}{\frac{L^{2}}{2w_{0}}+\frac{w_{0}}{2}}$$

where *R*’ is the radius of curvature, and *k*_02_ is the curvature of the bending plate.

#### 3.2.1. The Values of *w*_0_ and *L*

According to Equation (47), the key parameters to obtain a curvature are *w*_0_ and *L*. *w*_0_ can be obtained from Equation (44). If *L_p_* estimated by Equation (8) is larger than the thickness *h* of sheet, *L_p_* should be taken as *h*. After the material and laser parameter are chosen, *P_max_* can be estimated from Equations (12)–(14). The depth of plastic zone *L_p_*, and the impulse *I*, are related to the maximum pressure of plasma *P_max_*. According to the experiment, *L* = 3*R* is taken in this work, as shown in Figure 3.

It should be noted that, only satisfying the condition for obtaining the real solution of Equation (44), *w*_0_ can be derived. So, according to Equation (44),

(48) $$L_{P}>L_{th}$$

(49) $$L_{th}=h-\frac{3EI^{2}}{4k\rho \pi^{2}R^{2}L^{2}\sigma_{ym}^{2}}\cdot \frac{1}{h}$$

That is, SBM can induce concave curvatures only if the depth of laser-shock-induced plastic deformation reaches above the threshold value (*L_th_*), or else SBM should be disregarded.

#### 3.2.2. Determination of Average Stress Coefficient, *k*

In this case, taking the same laser energy ranges from 2 J to 6 J, if other process parameters are the same as those for convex bending 2024 aluminum alloys with a thickness *h*_1_ = 1 mm, the sheet will bend concavely. We measured the stresses along the depth direction of the 1 mm sheet when the laser pulse energy was 2 J. The stress magnitude of the bottom surface was about 40 MPa. Therefore, we took the stress coefficient *k* = 0.07 when the depth of plastic deformation *L_p_* estimated by Equation (8) exceeded the thickness of the sheet. Then, the third item of Equation (44) is zero and *k* is not needed.

### 3.3. The Total Curvature Due to the Combined Effects of SGM and SBM

As is given above, the bending curvature due to just SGM is *k*_01_, and that due to just SBM is *k*_02_. Actually, SGM and SBM coexist in laser peen forming and their combined effects determine the obtained deformation of targets, that is to say, *k*_0_ = *k*_01_ + *k*_02_. Under certain conditions, e.g., *L_p_* < *L_th_*, SGM dominates the deformation process. *k*_0_ = *k*_01_. On some other conditions, e.g., *L_p_* > *h*, SBM dominates during LPF. *k*_0_ = *k*_02_. Then, based on this principle, we can calculate the curvatures of bent sheet due to LPF. Table 1 gives the results for bending 3 mm, 2 mm, and 1 mm sheets induced by laser of 2 J, 4 J, 5 J, and 6 J.

### 3.4. Experimental Validation

**Figure 5:**
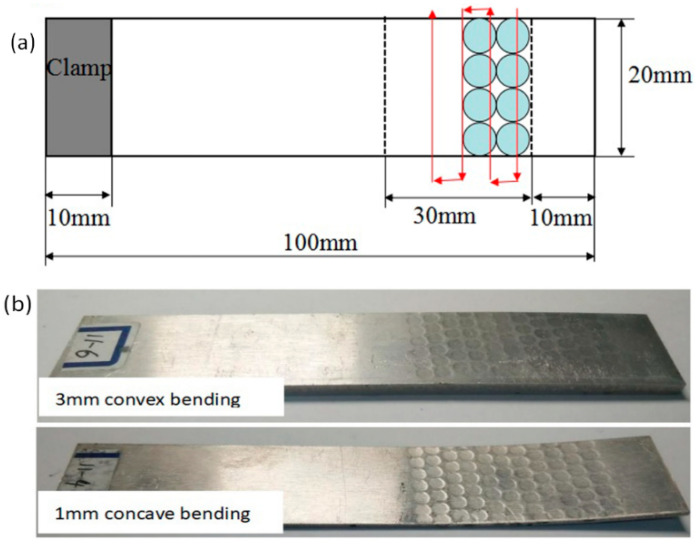
(**a**) Processing strategies of laser peen forming and (**b**) the typical results.

In order to validate the analytical model, a series of experiments were conducted using 2024 aluminum alloy plates with the same length and width but different thicknesses. The parameters of aluminum alloy plate are shown in Table 2. The length of plates was 100 mm, and the width was 20 mm. The plates had three different thicknesses: *h*_1_ = 1 mm, *h*_2_ = 2 mm, and *h*_3_ = 3 mm. Before laser peen forming, the plates were cleaned with alcohol. A 3M black tape of 0.1 mm thickness was placed on the plate surfaces as an absorbent layer. A water film acting as the confining layer and form a 2 mm thick flowing water film on the surface of the workpiece. The plates were clamped at one side like cantilever beams. A Q-switched Nd:YAG laser with a wavelength of 1064 nm was used. The laser pulse length was about 20 ns. The repetition rate ranged was 1 Hz, and laser pulse energy was between 2 J and 6 J. The radius of the circular laser beam spot with a uniform energy distribution was 1.5 mm, scanning speed was 3 mm/s, and number of scanning lines was 11. During the laser shock forming experiment, the laser beam impacted the surface along the normal of the surface as shown in Figure 5. After the laser peen forming, residual stress distributions along the depth direction were measured with an X-350A type X-ray stress meter. Figure 5 also shows typical convex and concave bending results.

**Figure 6:**
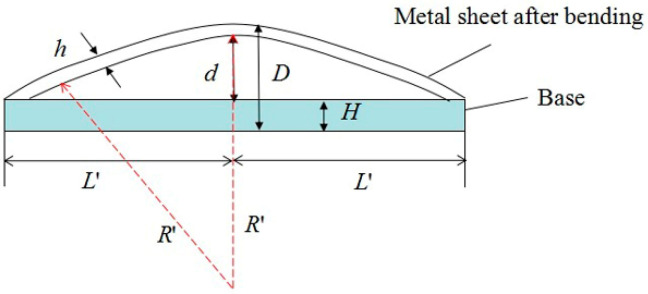
Geometrical schematic of a curvature.

The arc height of the deformed plates was measured, as shown in Figure 6, where *h* is the thickness of the bending plate. 2 mm thick 2024 aluminum alloy is chosen as the base, and its half-length is *L*’. *D* is the maximum height between the bending plate and the base, which can be easily measured. *d* is arc height, which equals to *D*-*H*-*h*. *R*’ is the approximate radius of bending plate, and *k*_0_ is the curvature. The curvature of the concave geometry is defined to be positive and that of the convex geometry is negative. The same base of *L*’ = 20 mm was used in this experiment. According to the geometry of the arc, one can deduce the following formula

(50) $$k_{0}=\frac{1}{\frac{{\dot{L}}^{2}}{2d}+\frac{d}{2}}$$

## 4. Results and Discussions

### 4.1. Convex Bending of 3 mm 2024 Aluminum Alloy

**Figure 7:**
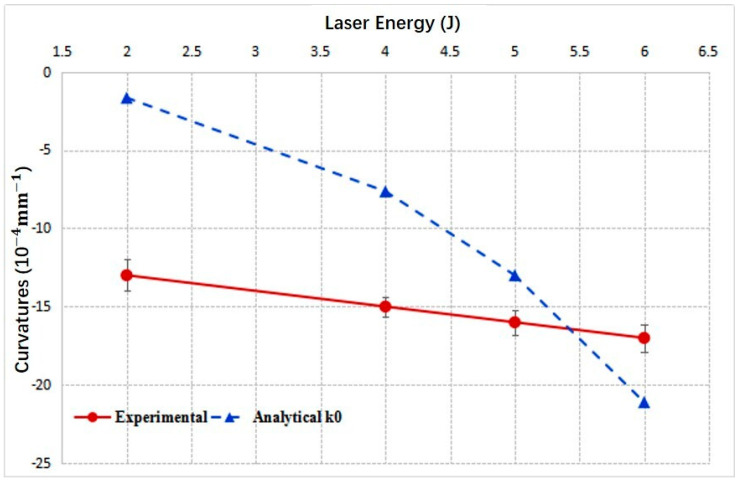
Curvatures of 3 mm 2024 aluminum alloy vs. laser pulse energies.

**Figure 8:**
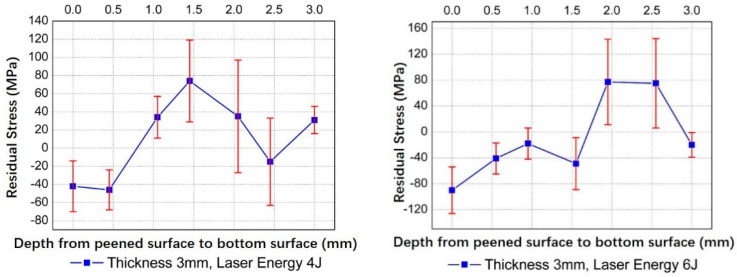
Stresses measured along the depth of 3 mm target for laser energies of 4 J and 6 J.

Figure 7 gives the curvatures vs. laser energies of convex bending for 3 mm thick 2024 aluminum alloy obtained through experiments and the analytical model. The trends of two lines are consistent, and the curvatures increase with the laser pulse energies. However, there still exist obvious deviations between them because the analytical model was developed based on many assumptions. As we have assumed, the stresses within the plastic layer were uniformly distributed along the depth direction. In reality, the stress distribution along depth direction may be complex. Figure 8 gives the measured residual stresses along the depth direction of the 3 mm sheet formed with laser energy of 4 J and 6 J. In addition, for the analytical results shown in Figure 7, the stress average coefficient *k* was set as a constant value of 0.1, while in the experiments, it might vary accordingly to changing laser energies. All these factors contributed to the deviations. Another phenomenon one can note is that the curvatures given by the analytical model increase more rapidly with increasing laser energy than the experimental results, especially for higher laser energy. During LPF, the final contours under all conditions must be the combined effects of SBM and SGM, although we have disregarded the concave bending effects in Table 1, due to *L_p_* < *L_th_*. At lower laser pulse energy, SGM dominates, while with increasing laser energy, the role of SBM played in forming the final contour becomes more and more important, although SGM still dominates the forming process. SBM will weaken the convex bending effect. Therefore, the actual experimental results with higher laser energy, e.g., 6 J in Figure 7, is smaller than those estimated with analytical model.

### 4.2. Concave Bending of 1 mm 2024 Aluminum Alloy

**Figure 9:**
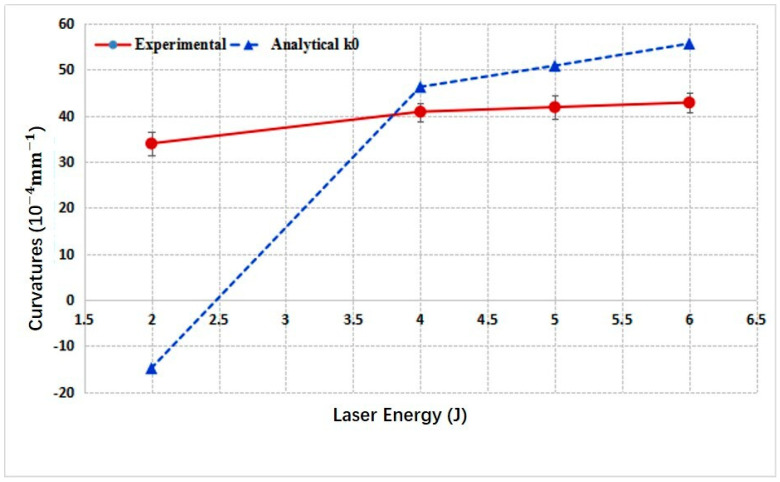
Curvatures of 1 mm 2024 aluminum alloy vs. laser pulse energies.

Figure 9 gives the curvatures vs. laser energies of concave bending obtained through experiments and the analytical model. The trends of two lines are consistent, and the curvatures increase with the laser pulse energy. Similar to Figure 7, curvatures increase more rapidly with increasing laser energies. It is because the final contours are the combined effects of SBM and SGM in the experiments. SGM will weaken the concave bending effect in the actual experiments. As the result, when laser energy increases, the concave curvatures increase moderately, rather than increase rapidly as predicted by analytical model. Additionally, from Figure 9 and Table 1, we can note that when laser energy is 2 J, SBM and SGM coexist, and SGM model overestimates the convex curvature *k*_01_. As the result, the analytical model gives the convex prediction finally, while in the experiment the metal sheet is concavely bent. In reality, we will find that for those conditions when SGM and SBM effects are comparable to each other, the analytical model developed will demonstrate the big predicting deviations, shown in the following context.

### 4.3. Convex Bending of 2 mm 2024 Aluminum Alloy

**Figure 10:**
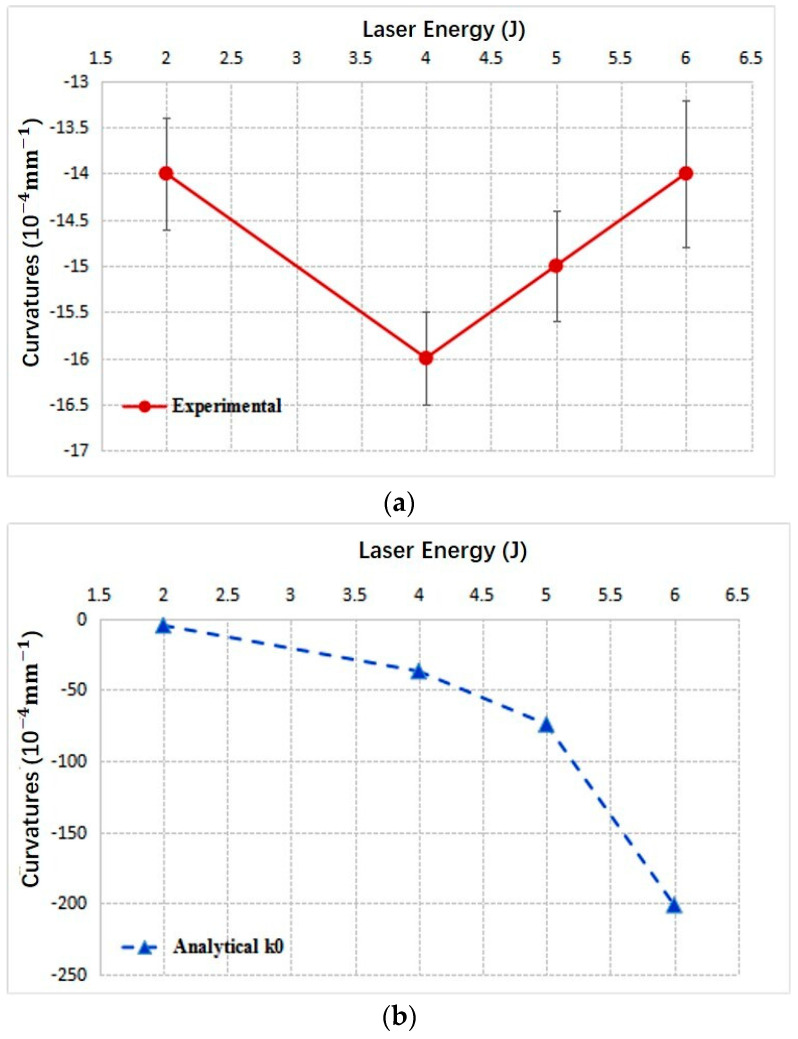
Curvatures of 2 mm 2024 aluminum alloy vs. laser pulse energies: (**a**) experiment (**b**) analytical model.

For the 2 mm samples, analytical model predicts the same bending direction as those of the experiments. However, except for laser energy 2 J, the analytical model gives so seriously deviated predicted values. The analytical results are nearly one order of magnitude larger than the experimental ones. Additionally, the trends of the experimental and analytical results are different. In Figure 10a, it is obviously observed that the convex curvatures increase firstly with increasing laser energy, reaching the maximum value at 4 J. Then, with further increasing laser energy, the convex curvatures decrease instead. However, curvature values predicted through the analytical model increase in a monotone manner. This deviation is mainly due to the convex curvature overestimation of the analytical model, especially for those conditions when SGM and SBM are comparable. In the experiment, when SGM overwhelmingly dominates, the curvatures of convex bending increase with laser pulse energy until reaching one certain energy, at which the effect of SBM not only offsets the increasing effect of SGM, but also begins to decrease the convex curvature of convex bending. For the 2 mm sample in our experiment, this turning point is at 4 J laser energy, as shown in Figure 10a. Although we did not conduct the experiments with the energies larger than 6 J because of the maximum energy limitation of our laser equipment, it can be expected that, if increasing laser energy further, the effect of SBM further intensifies, and the plate will turn from convex bending into concave bending. While for the analytical model, because of the overestimation of *k*_01_, seen from Table 1, the turning point did not appear in Figure 10b.

## 5. Conclusions

A combined analytical model was developed for estimating the bending curvatures after laser peen forming. The convex curvature *k*_01_ was predicted based on the internal force balance according to stress gradient mechanism, and the concave contribution *k*_02_ was developed based on the energy transformation. Their combined effects determine the obtained deformation of targets, that is to say, *k*_0_ = *k*_01_ + *k*_02._ When the depth of laser shock induced plastic deformation is below one certain threshold value (*L_p_* < *L_th_*), SBM should be disregarded. *k*_0_ = *k*_01_. Under some other conditions, e.g., *L_p_* > *h*, SBM dominates during LPF. *k*_0_ = *k*_02_. Experiments have been conducted to evaluate the developed model.

When just SGM or SBM overwhelmingly dominates the forming process, the analytical model can give relatively precise predicted curvatures comparing with those experimental ones. While under the conditions where both SBM and SGM effects are comparable, the accuracy of the analytical results is poor, because of the complex stress distributions within the material and the complex energy coupling process under these conditions. Improvements in the model can be made in the future to account for more complex parameter interactions in LPF.

## Figures and Tables

**Table 1:** Curvatures *k*_0_ of the target sheets calculated using the analytical model.

Targets		Laser Energies/J	2	4	5	6
	Curvatures/10^−4^ mm^−1^					
3 mm	*k*_01_		−1.67	−7.64	−13.02	−21.14
	*k*_02_		*L_p_* < *L_th_*, 0	*L_p_* < *L_th_*, 0	*L_p_* < *L_th_*, 0	*L_p_* < *L_th_*, 0
	*k*_0_		−1.67	−7.64	−13.02	−21.14
2 mm	*k*_01_		−4.79	−37.01	−88.71	−223.09
	*k*_02_		*L_p_* < *L_th_*, 0	*L_p_* < *L_th_*, 0	14.43	21.63
	*k*_0_		−4.79	−37.01	−74.28	−201.46
1 mm	*k*_01_		−45.85	*L_p_* > *h*, 0	*L_p_* > *h*, 0	*L_p_* > *h*, 0
	*k*_02_		31.01	46.29	50.87	55.71
	*k*_0_		−14.84	46.29	50.87	55.71

**Table 2:** Parameters of 2024 aluminum alloy.

Material	2024 Aluminum Alloy
Density *ρ*	2.7 g·cm^−3^
Elastic modulus *E*	72 GPa
Poisson’s ratio *ν*	0.34
Yield strength *σ_Y_*	290 MPa

## Data Availability

Data sharing not applicable.
